# Viewing the human microbiome through three-dimensional glasses: integrating structural and functional studies to better define the properties of myriad carbohydrate-active enzymes

**DOI:** 10.1107/S1744309110029088

**Published:** 2010-07-31

**Authors:** Peter J. Turnbaugh, Bernard Henrissat, Jeffrey I. Gordon

**Affiliations:** a FAS Center for Systems Biology, Harvard University, Cambridge, MA 02138, USA; b Architecture et Fonction des Macromolécules Biologiques, CNRS, Marseille, France; c Center for Genome Sciences and Systems Biology, Washington University School of Medicine, St Louis, MO 63108, USA

**Keywords:** human microbiome, carbohydrate-active enzymes

## Abstract

Recent studies have provided an unprecedented view of the trillions of microbes associated with the human body. The human microbiome harbors tremendous diversity at multiple levels: the species that colonize each individual and each body habitat; the genes that are found in each organism’s genome; the expression of these genes and the interactions and activities of their protein products. The sources of this diversity are wide-ranging and reflect both environmental and host factors. A major challenge moving forward is defining the precise functions of members of various families of proteins represented in our microbiomes, including the highly diverse carbohydrate-active enzymes (CAZymes) involved in numerous biologically important chemical transformations, such as the degradation of complex dietary polysaccharides. Coupling metagenomic analyses to structural genomics initiatives and to biochemical and other functional assays of CAZymes will be essential for determining how these as well as other microbiome-encoded proteins operate to shape the properties of microbial communities and their human hosts.

## Abstract 2

Metagenomics has unleashed a deluge of sequencing data describing the organismal, genetic, and transcriptional diversity of the human microbiome. To better understand the precise functions of the myriad proteins encoded by the microbiome, including carbohydrate-active enzymes, it will be critical to combine structural studies with functional analyses.

The human body is home to trillions of microorganisms, most of whom reside in our gastrointestinal tracts. A century ago, Arthur Kendall wrote

the multiplicity of types and variety of physiological requirements of this intestinal flora are … a strong reminder of the influence which the unrestrained activity of these organisms might conceivably exercise upon the general condition of the host

(Kendall, 1909 ▶). Fast-forward 100 years and culture-independent metagenomic studies are now generating a tsunami of DNA-sequence data characterizing the genetic components of our microbiomes. For example, recent reports have described the results of (i) surveying the microbial composition of 27 different body-habitat-associated communities within and between multiple individuals over time (Costello *et al.*, 2009 ▶); (ii) deeply sequencing the human gut microbiomes of genetically identical and unrelated individuals (Qin *et al.*, 2010 ▶; Turnbaugh *et al.*, 2010 ▶); (iii) analyzing ethnic variations in vaginal microbiomes (Ravel *et al.*, 2010 ▶) and (iv) producing reference databases composed of hundreds (and soon to be thousands) of genome sequences from cultured representatives of various human microbiota (Nelson *et al.*, 2010 ▶).

While these human microbiome projects (HMPs) are rapidly expanding the number of gene sequences being deposited in public databases, as is the case with the human genome project there are significant challenges in developing efficient and economical strategies for exploiting these data in order to gain insights into the functions of protein products and how they contribute to human physiology, physiological variations, disease risk and disease pathogenesis. Addressing these challenges requires an integration of many experimental approaches, ranging from functional metagenomics (Craig *et al.*, 2010 ▶; Uchiyama & Miyazaki, 2009 ▶; Sommer *et al.*, 2009 ▶, 2010 ▶) to high-throughput genetic screens to identify determinants of symbiont fitness in a given microbial community or host context (Goodman *et al.*, 2009 ▶) and structural genomics initiatives targeted to various groups of microbiome-specified proteins (see papers in this issue).

One issue that potentially confounds the design of microbiome-directed structural genomic ‘campaigns’ is the incredible amount of microbial organismal and genetic diversity found in communities occupying various human-body habitats. A recent deep sampling, using culture-independent metagenomic methods, of the fecal microbiota of an adult monozygotic female twin pair revealed an estimated 800–900 bacterial species in each co-twin, less than half of which were shared by both individuals (Turnbaugh *et al.*, 2010 ▶). Expanding this analysis to include a shallower sampling of 281 fecal samples obtained from 54 monozygotic and dizygotic twin pairs and their mothers revealed >4000 gut-associated species-level phylo­genetic types (phylotypes). However, of the 134 bacterial species whose relative abundance was >0.1% in at least one fecal community, only 37 were detected in more than half of the biospecimens collected in this survey of ∼150 individuals.

Deep shotgun pyrosequencing of fecal community DNAs prepared from the monozygotic twin pair referred to above yielded >100 000 protein-coding gene clusters. Only 17% of the clusters identified in this combined 10.1 Gbp data set were shared between these ‘genetically identical’ individuals; 49% encoded hypothetical proteins (no assignable COG, GO-term, TIGRfam or Pfam annotations) and only 36% had significant matches to known or predicted proteins present in the genomes of 122 cultured members of the human gut microbiota (Turnbaugh *et al.*, 2010 ▶).

Annotation of the genes identified in this deeply sampled gut-microbiome data set using the carbohydrate-active enzymes (CAZy) database (Cantarel *et al.*, 2009 ▶) disclosed 143 different CAZy families encoded by 5145 genes (Turnbaugh *et al.*, 2010 ▶). The revealed CAZyme repertoire emphasizes two themes emerging from present-day explorations of the human gut microbiome: substantial interpersonal variation and the disclosure of unanticipated functions. For example, genomic segments from the genus *Faecalibacterium* in the microbiome of one co-twin contained a number of genes encoding predicted cellulases [members of glycoside hydrolase family 5 (GH5), GH9, GH44 and GH48] plus 42 genes encoding putative dockerins (small proteins that aid in the assembly of extracellular cellulosomes; Bayer *et al.*, 2008 ▶). These genes were notably absent in her co-twin’s microbiome or in a draft genome assembly produced from *F. prausnitzii* strain M21/2. Pyrosequencing reads homologous to predicted dockerins were found in 18 other human fecal microbiomes, representing six sets of adult monozygotic twins and their mothers, but varied in their abundance between individuals within and between families (Turnbaugh *et al.*, 2010 ▶).

Tallying the results of the metagenomic analysis of these gut-microbiome data sets from families containing monozygotic twins has so far yielded 156 CAZy families (including 77 glycoside hydrolase, 21 carbohydrate-binding module, 35 glycosyltransferase, 12 polysaccharide lyase and 11 carbohydrate esterase families). This means that CAZymes represent on average 2.6% of the sequenced genes in each microbiome (Turnbaugh *et al.*, 2009 ▶). In contrast, the human genome encodes at best 20–25 digestive enzymes from CAZy families GH1 (lactase), GH13 (α-amylase) and GH31 (maltase, isomaltase and sucrase). Thus, with the exception of starch and sucrose, our ability to digest dietary plant carbohydrates resides entirely in our gut microbiomes.

The CAZymes represented in different human populations consuming different diets may be influenced by their varied cultural traditions. This point is illustrated by a recent study that started out by investigating the polysaccharide-degrading capabilities of *Zobellia galactanivorans*, a marine Bacteroidete that can metabolize porphyran derived from marine red algae belonging to the genus *Porphyra* (Hehemann *et al.*, 2010 ▶). Porphyranases from *Z. galactani­vorans* were isolated, their biochemical activities were confirmed and the structures of two of them were determined by X-ray crystallography (PDB entries 3juu and 3ilf; Hehemann *et al.*, 2010 ▶). Homologous genes were found in the common human gut bacterium *Bacteroides plebeius*. Intriguingly, these genes were represented in the gut microbiomes of Japanese but not North American individuals. These findings are consistent with a horizontal gene-transfer event whereby porphyranases from an ancestral marine bacterium related to the Bacteroidetes *Z. galactanivorans* and *Microscilla* sp. PRE1 were acquired by a resident member of the gut microbiota of Japanese consumers of nonsterile food; this microbiome-acquired trait may have then been disseminated to other members of this human society.

Systematic application of high-throughput structural genomics initiatives to CAZymes represented in human microbiomes will undoubtedly yield protein folds together with the expectation that new insights/predictions about their functions will ensue. However, proteins that interact with complex carbohydrates pose some difficult challenges when trying to elucidate function ‘just’ through fold determination. The reason relates at least in part to the fact that these proteins must selectively recognize and process one of the most diverse classes of biological substrates on Earth: substrates that possess an enormous range of stereochemical and structural variations (Laine, 1994 ▶). These variations in turn are harnessed by living organisms to fulfill very different roles: *e.g.* structural, storage, specific signaling, specific recognition among myriad similar molecules, host–pathogen interactions and exchanges between symbionts to name but a few. Predictions such as ‘putative glycoside hydrolase’ or ‘putative carbohydrate-binding protein’ are still a long way from what is desirable, owing to the different functions that carbohydrates achieve depending on seemingly small structural variations. A vivid example is provided by polymers of d-glucose residues linked between C atoms 1 and 4: if the glycosidic bond is α the polymer is amylose and is used for carbon storage by most cells; if the glycosidic bond is β the polymer is cellulose, one of nature’s toughest structural polysaccharides.

Carbohydrate diversity greatly exceeds the number of known protein folds and is undoubtedly one reason for the observed diversity of genes encoding putative CAZymes (Fig. 1 ▶ *a*). One consequence is that gene families group together enzymes with widely different substrate or product specificities (Henrissat, 1991 ▶). Another consequence is that to derive knowledge useful for subsequent functional predictions, we must adopt a divide-and-conquer approach whereby subgroups (subfamilies, clusters) are defined within families and then functional characterization in each subgroup without a biochemically established member is performed. This process needs refinement, as the appropriate threshold for defining a subfamily is likely to vary from one family to another. For example, there are extreme cases such as blood-group transferases where two amino-acid changes switch the substrate specificity for the transferred carbohydrate from galactose to *N*-acetylgalactosamine, thereby changing the resulting blood-group epitope from B to A (Seto *et al.*, 1999 ▶). At the other end of the spectrum, cellulases with as little as 10% sequence identity can digest the same substrate (Henrissat *et al.*, 1989 ▶).

Microbes are particularly ingenious at inventing novel CAZymes, either from other CAZymes or from other ‘pre-existing’ scaffolds; *e.g.* at least five different folds are known for cellulases (1cec, 1cb2, 1cel, 1clc, 2eng; Dominguez *et al.*, 1995 ▶; Koivula *et al.*, 1996 ▶; Divne *et al.*, 1994 ▶; M. B. Lascombe, H. Souchon, M. Juy & P. M. Alzari, unpublished work; Davies *et al.*, 1995 ▶). CAZymes have a highly variable modular structure, with the catalytic module carrying a variable number of ancillary modules. Each module can provide a complementary catalytic activity or carbohydrate-binding, protein-binding, cell-binding (or other unknown) capabilities. The number of domain combinations is very large and this is one way devised by living organisms to increase the number of carbohydrate-interacting proteins using a limited number of folds. A consequence of this modular variability (Fig. 1 ▶ *b*) is that the precise function of a module sitting next to a carbohydrate-binding module (CBM) is hard to predict since it could be catalytic (*e.g.* glycoside hydrolase, polysaccharide lyase, carbohydrate esterase, protease or kinase) or noncatalytic (*e.g.* another CBM, a membrane-attachment domain or a module involved in cell adhesion or in cellulosome assembly). Some carbohydrate-interacting proteins are made of a single CBM, while others may contain two noncatalytic modules (*e.g.* CBM33-CBM2; expansins). Syntrophy (cross-feeding) between any two organisms can be conceptualized as the proportion of ‘seeds’ (molecules acquired exogenously from the environment; Borenstein *et al.*, 2008 ▶) that are intermediates or end products in another, while competition can be viewed as the number of seeds that are shared. Microbes need to remain in the vicinity of their seeds (substrates) to establish and maintain syntrophic relationships and it is entirely possible that some dual binding proteins exist to bind a carbohydrate on the one hand and some component of a neighbor’s cell surface on the other.

One class of CAZymes, the glycosyltransferases (GT), are particularly difficult to study experimentally owing to their frequent association with membranes, their poor stability in pure form and their low solubility (Davies *et al.*, 2005 ▶). Assays for enzymatic function are also difficult because the nucleotide diphospho-sugar donor must be identified along with the acceptor, which could potentially span a wide range of molecules. In addition, GTs may lack great specificity *in vitro*. This observation can be rationalized by the fact that GTs frequently encounter a narrow set of donor/acceptor molecules by virtue of their compartmentalization within cells or because metabolic fluxes deliver the ‘appropriate’ reagents to these enzymes. Moreover, the few structural studies of GTs reported to date have revealed that they undergo important conformational changes upon ligand binding and that only the ternary complex with both donor and acceptor achieves the conformation suitable for catalysis (Lairson *et al.*, 2008 ▶). While significant success has been achieved by structural genomics initiatives with proteins that bind or break down complex carbohydrates, the number of GT structures remains comparatively modest (Table 1 ▶).

Even if a structure is solved for a putative glycoside hydrolase, it can be difficult to infer its function. One way around this problem is to determine whether there are conserved aspartate or glutamate residues that are suitably positioned to perform catalysis (McCarter & Withers, 1994 ▶; Davies & Henrissat, 1995 ▶, 2002 ▶). Although the catalytic residues of GHs are usually strictly conserved, there are examples that serve as a warning against over-interpretation of *in silico* predictions and as a strong incentive for rigorous experimental characterization (Table 2 ▶). Currently, there are several active collaborative efforts for research and education in the glycosciences both in the USA and Europe; *e.g.* the Consortium for Functional Glycomics (http://www.functionalglycomics.org/), the Euroglycosciences Forum (http://www.egsf.org/) and the German Glycosciences Initiative (http://www.glycosciences.de/). These resources could be expanded and/or used as starting points for additional efforts aimed at systematic functional characterization of carbohydrate-active enzymes identified by mining the massive data sets emanating from the ever-increasing numbers of metagenomic studies of microbial communities.

In summary, for CAZymes, CBMs and other sugar-interacting proteins, perhaps more than for any other protein class, there is an enormous need to join metagenomics together with structural biology initiatives and to link the output with functional assays. In this respect, the remarkable structures of microbiome-encoded proteins that are being solved through structural genomics initiatives represent islands in a sea of insecure predictions. Follow-up biochemical work needs to be performed to identify the actual carbohydrate structures recognized and processed by a given protein whose fold has been characterized. Accelerating the pace of discovery of these carbohydrate structures will require new experimental innovations, as well as the sponsorship of individuals who wish to train in the glycosciences.

## Figures and Tables

**Figure 1:**
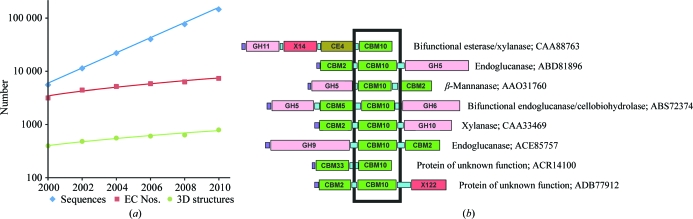
CAZymes are modular and increasingly described only by high-throughput sequencing data. (*a*) Growth of sequence, functional and structural data in the CAZy database. (*b*) Example of modular variation in CAZymes containing a common CBM10 domain.

**Table 1:** Proportion of functionally and structurally characterized glycoside hydrolases (GHs), polysaccharide lyases (PLs) and glycosyltransferases (GTs) in the CAZy database as of June 2010

	GHs	PLs	GTs
Entries with assigned enzyme-classification numbers (%)	6.7	11.0	2.9
Entries whose structures have been determined (%)	0.75	2.4	0.17

**Table 2:** Examples of ‘misleading’ CAZymes or CAZyme-like proteins

Protein	Description	Reference
Myrosinase	A plant sugar-cleaving enzyme that has evolved from GH1 β-­glucosidases by losing one of the two catalytic Glu residues, which has been replaced by an ascorbate cofactor.	Burmeister *et al.* (2000 ▶)
α-Lactalbumin	Noncatalytic protein; shares 40% sequence identity with type C lysozymes; would be predicted to be a lysozyme based on sequence similarity.	Brew *et al.* (1967 ▶)
Wheat xylanase inhibitor protein XIP-I	A xylanase inhibitor that displays sequence and structural similarity to chitinases, but has nonetheless lost its catalytic activity. Many orthologs are still annotated in GenBank as putative chitinases (for example, GenBank BAC10141).	Payan *et al.* (2004 ▶)
Glycosidase family 97 proteins (GH97)	This family contains two subgroups, each with different catalytic machinery and stereochemical outcome, making it difficult to define the catalytic machinery based on invariant acidic residues.	Gloster *et al.* (2008 ▶)
Glycosidase GH4 and GH109 proteins	Proteins from these families would be classified as NAD oxido­reductases based on sequence and structure only.	Liu *et al.* (2007 ▶)
